# A Quantitative Analysis of Human and Material Resources for Endoscopy Services in Pacific Island Countries

**DOI:** 10.1002/jgh3.70068

**Published:** 2024-12-12

**Authors:** Mai Ling Perman, Chris Hair, Joji Malani, Finlay Macrae, Dianne Jones, Eileen Natuzzi, Rooney Jagilly

**Affiliations:** ^1^ Fiji National University Suva Fiji; ^2^ Australian and New Zealand Gastroenterology International Training Association Melbourne Australia; ^3^ Deakin University Waurn Ponds Victoria Australia; ^4^ Royal Melbourne Hospital Melbourne Australia; ^5^ Walsh School of Foreign Service, Center for Australia, New Zealand and Pacific Studies Georgetown University Washington DC USA; ^6^ Ministry of Health Honiara Solomon Islands

**Keywords:** capacity, endoscopy, gastroenterology, Pacific, training

## Abstract

**Aims:**

This study aims to evaluate the current state of endoscopy services in Pacific Island Countries (PICs) by quantifying human and material resources, including the number of trained endoscopists and nurses, the types of procedures performed, and the availability and maintenance of endoscopic equipment.

**Methods and Results:**

A mixed methods survey was conducted in 2023, targeting doctors and nurses who participated in the WGO‐FNU‐ANZGITA endoscopy training program as well as non‐participants. Survey invitations were sent through email, WhatsApp, and Facebook Messenger. Data were collected from 16 sites across 12 PICs, achieving an 85% response rate. Survey results indicated a total of 58 endoscopists (2.1/100000 population) and 52 nurses (1.9/100000 population), with a majority having received training through international partnerships. Basic endoscopy services, such as gastroscopy and colonoscopy, were widely available, but advanced procedures were limited to a few sites. Most sites reported using donated equipment, with significant challenges in equipment maintenance and repair. The availability of functional endoscopes averaged four per site. Common issues included outdated equipment, lack of qualified personnel, and insufficient funding for new equipment.

**Conclusion:**

Despite efforts to enhance endoscopy services in PICs through international collaborations, significant gaps remain, particularly in terms of advanced procedural capacity and equipment maintenance. Recommendations include expanding training programs, improving equipment maintenance infrastructure, securing funding for new equipment, and fostering stronger partnerships to support the sustainability of endoscopy services. Addressing these areas can enhance the quality and availability of endoscopy services, ultimately improving healthcare outcomes for populations in PICs.

**Abbreviations**
CWM: Colonial War Memorial Hospital
FSM: Federated States of Micronesia
RMI: Republic of Marshall Islands

## 1 Introduction

The provision of adequate healthcare services, including diagnostic and therapeutic procedures such as endoscopy, is a component of the Universal Healthcare Coverage, which is crucial for ensuring the well‐being of populations in Pacific Island countries (PIC). The Pacific Islands are a diverse group of nations scattered across the vast expanse of the Pacific Ocean, facing unique socio‐economic and healthcare challenges [1, 2]. PIC have considerable diversity in size, population, ethnicity, income status, and health outcomes [3, 4]. Despite efforts to improve healthcare infrastructure and services, disparities persist, particularly with regard to the availability of specialized medical procedures such as endoscopy.

Endoscopy plays a pivotal role in the diagnosis and management of various gastrointestinal disorders, including cancer, ulcers, infectious, and inflammatory conditions. PIC face common barriers to endoscopy including resource constraints, geographical isolation, limited skilled workforces, distance from the global economy, and vulnerability to climate change [5, 6, 7, 8].

Since 2009, active efforts to enhance education and training in gastroenterology and endoscopy have occurred in a centralized training hub in Suva, Fiji in a collaboration between members of the Fiji National University (FNU) and Fiji Ministry of Health, the World Gastroenterology Organisation (WGO) and the Australian and New Zealand Gastroenterology International Training Association (ANZGITA) [9, 10]. Furthermore, several PIC have received in‐country support for education and development with ANZGITA and other partnerships [11]. These initiatives have seen endoscopy services established in many PIC, but there is a lack of data regarding the current endoscopy activities and resources required to sustain these services. The assessment of human and material resources for endoscopy services in PIC is not only vital for identifying deficiencies, but also for highlighting areas of strength and potential collaboration. By elucidating the existing capacity and challenges faced by PIC endoscopy services, improvement in partnerships between local authorities, international organizations, and other stakeholders may assist to bolster endoscopy services ultimately improve healthcare outcomes for the populations of PIC.

This study aims to address the paucity of data regarding the current activities of endoscopy services, and the human and material resources allocated to maintain these services in PIC by quantifying the current state of these resources, including the number of trained endoscopists and nurses, type of activity provided, the availability of endoscopic equipment, and infrastructure support.

## 2 Methods

An online mixed methods survey to evaluate the capacity of PIC to provide endoscopy services was conducted in 2023. Survey invitations were sent to doctors and nurses who had participated in the WGO‐FNU‐ANZGITA endoscopy training program at Colonial War Memorial Hospital in Suva, Fiji as well as to doctors and nurses belonging to professional organizations in the region regardless of whether they participated in WGO‐FNU‐ANZGITA endoscopy training. Surveys and consent forms were sent to senior clinicians using email, WhatsApp, and Facebook Messenger. Where a direct contact was unavailable an email requesting a contact person was sent to the country's permanent secretary of health. Private facilities were included where present. Reminders were sent to all participants at 3 week intervals. The survey instrument was developed and executed using SurveyMonkey, (San Mateo, California, USA). Participants answered a 61 question survey that included multiple choice answers as well as open text responses. The survey questions can be found in the Supplemental information.

Study approval was obtained from the Human Health Research Ethics Committee at Fiji National University, Suva, Fiji and at each participating site.

Data from each respondent were assigned an ID number and anonymously collected using Excel spreadsheets. Multiple respondents at the same facility site were merged and data analyzed by site and country. Data analysis included descriptive statistics on basic endoscopy, therapeutic or interventional endoscopy, type of equipment, and workforce. Each site had at least one nurse and one physician or surgeon reporting.

Site mean values were used in reporting endoscopies performed using categories. Non‐responding countries and countries not doing endoscopy were not included in the data analysis. Where there were multiple respondents at the same site, means were calculated along with value ranges. Conflicting responses were resolved using physician responses for procedure‐related questions and nurses' responses related to the care of the endoscopes and supplies.

Endoscopy procedures were reported using population‐based standardization by site or country. Population data were obtained from the World Bank 2022. Procedures per 1000 population were calculated.

## 3 Results

### 3.1 Demographics of Survey Respondents

Of 21 Pacific Island sites sent the survey, we received responses from 18 sites in 14 PIC (Table 1) for a response rate of 85%. Two respondents, Tokelau and Niue, did not participate in the survey as they do not have endoscopy services. Of the 16 sites with endoscopy services that responded, 10 respondents were non‐surgeon doctors, 12 were surgeon doctors, 2 were trainees or registrars and 17 were nurses. Table 1 shows the population served by each respondent site as well as the country's health expenditure per capita; 94% of sites reporting were in public hospitals.

**TABLE 1:** Numbers of respondents to the survey by country, type of health provider (nurse, non‐surgeon or surgeon doctor, and trainee), and public versus private classification of site. Multiple sites within a country are included separately.

Country (*n* = 16)	Respondents (*n* = 39)	Respondent site	Population 2022 World Bank	Health expenditure per capita $US WHO 2021	Public vs. private	Endoscopy available	Equipment brand
American Samoa	1 Nurse 1 Non‐surgeon doctor	LBJ Tropical Medical Center	44 000	692	Public	Gastroscopy Colonoscopy	Olympus
Cook	1 Nurse 1 Surgeon doctor	Rarotonga Hospital	17 000	737	Public	Gastroscopy Colonoscopy	Pentax
FSM Chuuk	1 Nurse 1 Surgeon doctor	Chuuk State Hospital	46000*	393	Public	Gastroscopy Colonoscopy	Fujinon
FSM Pohnpei	1 Nurse 1 Non‐surgeon doctor	Pohnpei State Hospital	39000*	393	Public	Gastroscopy Colonoscopy	Pentax
Fiji Lautoka	1 Nurse 2 Non‐surgeon doctor 1 Trainee	Lautoka Aspen Medical Hospital	80000**	250	Public	Gastroscopy Colonoscopy	Olympus
Fiji Labasa	1 Nurse 1 Non‐surgeon doctor 1 Surgeon doctor	Labasa Hospital	160000**	250	Public	Gastroscopy Colonoscopy	Pentax Olympus
Fiji CWM	1 Nurse 1 Non‐surgeon doctor	Colonial War Memorial Hospital	97000**	250	Public	Gastroscopy Colonoscopy ERCP	Fujinon
Fiji Private	1 Surgeon doctor	Pacific Specialist Healthcare Hospital	97000**	250	Private	Gastroscopy Colonoscopy	Huger
Kiribati	1 Nurse 1 Surgeon doctor	Tungaru Central Hospital	131 000	262	Public	Gastroscopy Colonoscopy	Pentax Olympus
Marshall Islands	1 Nurse 1 Surgeon doctor	Majuro Hospital	42 000	767	Public	Gastroscopy Colonoscopy	Olympus
Palau	1 Nurse 1 Non‐surgeon doctor	Belau National Hospital	18 000	2045	Public	Gastroscopy Colonoscopy	Fujinon
Samoa	2 Nurses 3 Surgeon doctors 1 Trainee	Tupua Tamasese Meaole Hospital	222 000	264	Public	Gastroscopy Colonoscopy	Olympus Fujinon
Solomon Islands	1 Nurse 1 Surgeon doctor	National Referral Hospital	724 000	106	Public	Gastroscopy Colonoscopy	Pentax
Solomon Islands	1 Nurse 1 Surgeon doctor	Gizo Provincial Hospital	200 000	106	Public	Gastroscopy Colonoscopy	Olympus
Tonga	1 Nurse 1 Non‐surgeon doctor	Vaiola Hospital	107 000	279	Public	Gastroscopy Colonoscopy	Olympus
Tuvalu	1 Nurse	Princess Margaret Hospital	11 000	1071	Public	Gastroscopy Colonoscopy	Huger
Vanuatu	1 Nurse 2 Non‐surgeon doctor 1 Surgeon doctor	Vila Central Hospital	327 000	133	Public	Gastroscopy Colonoscopy	Pentax Olympus

* Total population FSM 11 400.

** Total population Fiji 930 000.

**FIGURE 1:**
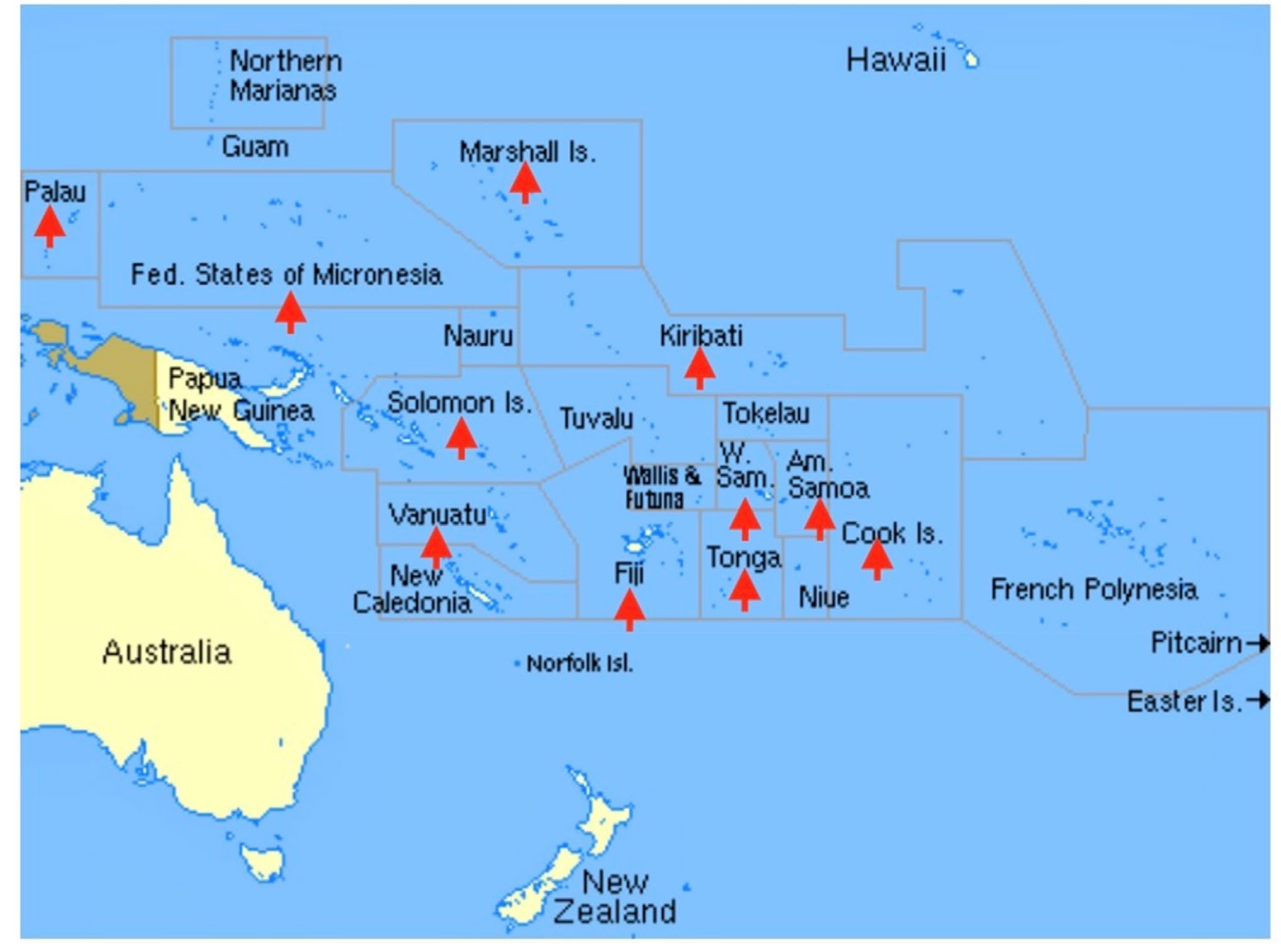
Sites where endoscopy is provided in 12 Pacific Island Countries. Red arrows denote where endoscopy is provided. Endoscopy is provided in more than one location in the Federated States of Micronesia, Fiji, Solomon Islands, Tonga and Vanuatu for a total of 17 sites where endoscopy is performed. *Source:* Network Startup Resource Center. https://nsrc.org/regions/OCEANIA/ .

Five sites reported having additional locations in their country where endoscopy is provided either by a dedicated service or by visiting teams (Figure 1). Fiji, Solomon Islands, and Federated States of Micronesia have more than one hospital where basic endoscopic procedures are done. Vanuatu and Tonga provide periodic endoscopy during visits to secondary hospital sites.

Briefly, 75% of respondent sites performed endoscopy in the operating theater. Six sites had a designated hospital endoscopy unit with half of those also using the operating theater as needed. The manufacturers of the gastroscopes and colonoscopes most frequently used by respondents include Olympus 50%, Pentax 38%, Fujinon 19%, Huger 6%; 25% of endoscopy sites report using equipment from multiple manufacturers with the most common pairing being Olympus and Pentax.

### 3.2 Endoscopy Workforce and Training

The total number of endoscopists reported (surgeons and physicians combined) was 58, representing a rate of 2.1/100000 population (Table 2). In 4 countries, only one endoscopist was available (Tuvalu, Pohnpei, Republic of Marshall Islands, and Palau). The total number of nurses working in endoscopy was 52 for the region representing a rate of 1.9/100000 population; 94% of the nurses working in endoscopy units are operating theater nurses and split their time between endoscopy and surgery. 94% of endoscopy sites have an endoscopy lead nurse with on average 7.5 years of experience.

**TABLE 2:** Endoscopy unit workforce including surgeons, non‐surgeon doctors, trainees, and nurses.

Country	Number endoscopists	Surgeon	Internal medicine physician	Gastrointestinal specialist	Registrar/doctor in training	Nurses*	Lead nurse
American Samoa	3	2	1	0	0	4	Yes
Cook	2	2	0	0	0	3	Yes
FSM Chuuk	2	2	0	0	0	4	Yes
FSM Pohnpei	1	0	1	0	0	1	Yes
Fiji Lautoka	7	4	3	0	2	3	Yes
Fiji Labasa	5	3	2	0	4	1	Yes
Fiji CWM	9	5	4	3	5	3	Yes
Fiji Private	3	3	0	0	0	1	No
Kiribati	3	2	1	0	1	5	Yes
Marshall Islands	1	1	0	0	0	4	Yes
Palau	1	0	1	0	0	6	Yes
Samoa	5	3	2	0	6	10	Yes
Solomon Islands	5	3	2	0	3	4	Yes
Tonga	6	3	3	0	3	1	Yes
Tuvalu	1	1	0	0	0	1	Yes
Vanuatu	5	3	2	0	0	1	Yes
Total	58	37	22	3	24	52	

* Nurses provide care and monitoring of patients during endoscopy, as well as cleaning and processing the equipment. There are no nurse anesthetists currently working in the Pacific Islands.

**FIGURE 2:**
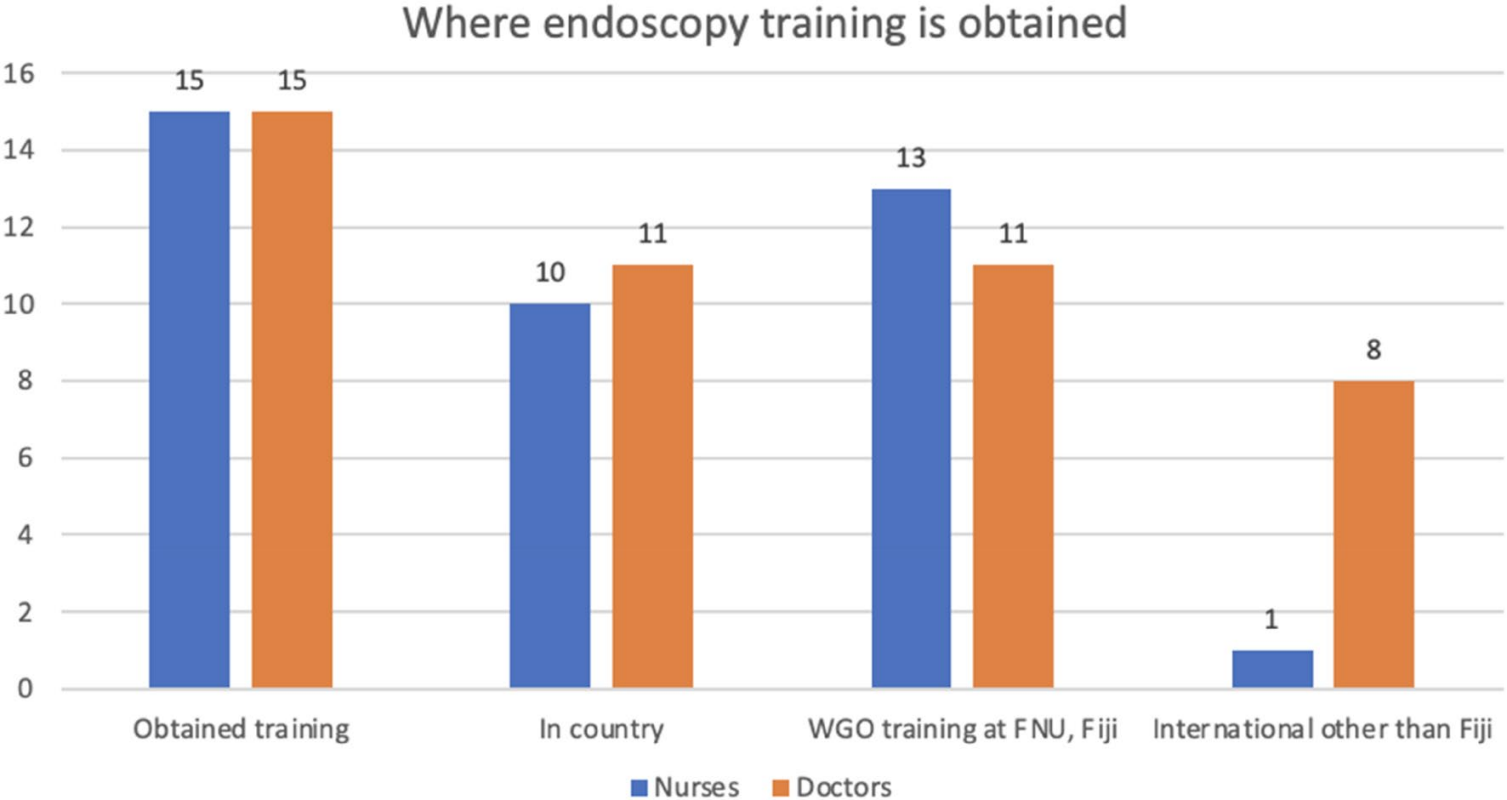
Where Pacific Island endoscopists and nurses receive training on performing endoscopic procedures, monitoring patients, and maintaining equipment.

Figure 2 shows where endoscopy training was obtained. The majority of nurses as well as doctors obtained their training through the WGO‐FNU‐ANZGITA endoscopy training scheme.

### 3.3 Endoscopic Procedures Performed

Briefly, 15 of 16 sites reported providing gastroscopy and colonoscopy. One site has the equipment available to do endoscopy but has not initiated endoscopy as yet (Tuvalu). 43% of sites reported doing flexible sigmoidoscopy, and 43% of sites reported doing rigid sigmoidoscopy. None of the sites do enteroscopy. Colonial War Memorial Hospital in Suva, Fiji is the only endoscopy site that provides capsule endoscopy and ERCP.

**FIGURE 3:**
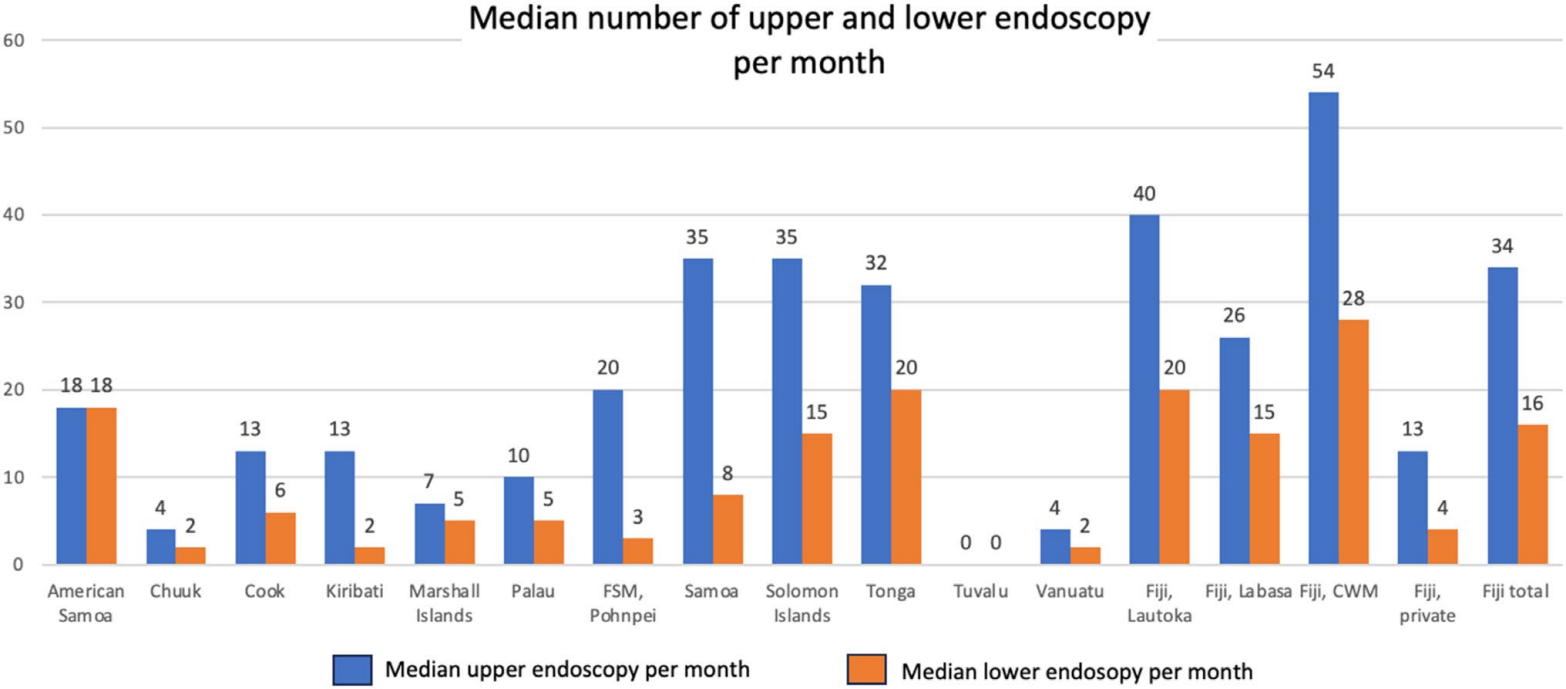
Median monthly rates of upper and lower endoscopy performed at responding PIC sites.

The median number of upper and lower endoscopies per month (Figure 3) varied by location and by procedure. Rates of gastroscopy were consistently higher than colonoscopy rates likely reflecting more technical challenges in learning to perform colonoscopy. The annual rate of endoscopy per 1000 population is in Figure S1.

Availability of basic endoscopy services is listed in the WHO Universal Healthcare Coverage Compendium of Care [12] and advanced endoscopy services that should be available at referral hospitals are shown in Table 3. No sites offered sclerotherapy for GI bleeds or varices, and clips or thermal probe treatments were employed less frequently. Only 50% of programs reported having a working diathermy unit. The majority of advanced endoscopic procedures reflect the two sites where endoscopy training has been heavily focused: Fiji and Solomon Islands.

**TABLE 3:** Upper endoscopy basic and advanced interventional services available including volume of upper and lower endoscopy per month. ✓ designates procedure is done in country.

	Average no. procedures/month		Basic procedures							Advanced procedures				
	Upper	Lower	Polypectomy	Biopsy	Inject bleeder	Thermal probe	Variceal banding	Clip placement	Foreign body retrieval	ERCP	Esophageal therapies			PEG
											Stricture dilatation	Achalasia	Stent	
American Samoa	15–20	15–20	✓	✓	✓	✓		✓	✓					✓
Cook	10–15	6	✓	✓					✓					
FSM Chuuk	2–5	2		✓	✓				✓			✓		
FSM Pohnpei	20	3	✓	✓	✓						✓			
Fiji Lautoka	30–50	20	✓	✓	✓		✓	✓	✓		✓	✓	✓	✓
Fiji Labasa	26	15	✓	✓	✓				✓		✓	✓	✓	✓
Fiji CWM	54	28	✓	✓	✓	✓	✓	✓	✓	✓	✓	✓	✓	✓
Fiji Private	10–15	3–5	✓	✓					✓					
Kiribati	10	2		✓	✓		✓				✓			
Marshall Islands	6–8	5	✓	✓	✓			✓	✓					
Palau	10	5	✓	✓		✓	✓	✓	✓					
Samoa	20–50	6–10	✓	✓			✓	✓	✓					✓
Solomon Islands	35	15	✓	✓		✓	✓	✓	✓		✓		✓	✓
Tonga	32	20		✓	✓		✓	✓	✓		✓	✓		
Tuvalu	0	0												
Vanuatu	3–4	2		✓	✓		✓	✓	✓					

### 3.4 Endoscopic Equipment, Maintenance, and Care

Table 4 summarizes the number of functional and nonfunctional gastroscopes and colonoscopes as well as where repairs are done and how scopes are obtained. The average number of functional endoscopes reported was 4 per site, with an average of 2 gastroscopes and 2 colonoscopes. How endoscopy equipment were obtained varies. The majority of programs (75%) send endoscopes overseas for repair either based upon a service contract or independently. Six respondent sites relied on donation alone, and 3 on a combination of donations and purchase through the Ministry of Health budget. Endoscopy equipment at 2 sites was purchased by the hospital or facility (American Samoa and Fiji private hospital) and one site, Marshall Islands, purchased endoscopy equipment through both the MOH as well as through their clinic or hospital.

**TABLE 4:** The total number of endoscopes, number of functioning gastroscopes and colonoscopes, and the number of scopes needing repair. How nonfunctioning scopes are repaired and how new endoscopes are acquired.

Country	Total endoscopes	Functional gastroscopes	Functional colonoscopes	Need repair	Maintenance	How equipment is obtained
American Samoa	5	3	2	0	Overseas repair	Purchased by facility
Cook	6	2	4	0	Service contract overseas	Purchased by MOH
FSM Chuuk	2	1	1	0	None available	Purchased by MOH
FSM Pohnpei	3	1	1	1	None available	Donation
Fiji Lautoka	4	1	2	1	Overseas repair	Donation purchased by MOH
Fiji Labasa	4	2	1	1	Overseas repair	Donation
Fiji CWM	12	4	3	5	Overseas repair	Donation
Fiji Private	2	1	1	0	Overseas repair	Purchased by facility
Kiribati	3	2	1	0	Overseas repair	Donation
Marshall Islands	2	1	1	0	Service contract overseas	Purchased by MOH Purchased by facility
Palau	4	1	3	0	Overseas repair	Purchased by MOH
Samoa	4	2	2	0	Overseas repair	Donation purchased by MOH
Solomon Islands	21	3	5	13	Overseas repair	Donation Purchased by MOH
Tonga	8	4	4	0	Overseas repair	Donation
Tuvalu	2	1	1	0	Do not know	Purchased by MOH
Vanuatu	3	1	2	0	None available	Donation

Responding sites reported using both reusable and single‐use disposable endoscopy supplies such as biopsy forceps. Most of the responding sites report they reuse some single‐use supplies such as biopsy forceps. Table S1 lists the types of single‐use items available at respondent sites. Eleven (69%) of sites use a combination of single‐use and reusable supplies, 19% use only single‐use supplies. Nine sites use variceal banding kits. Disposal of endoscopy supply waste is handled by the hospital medical waste system. Three respondents sites reported medical waste is incinerated and one reported medical waste is buried.

All respondent sites report endoscopes are processed by the nursing staff (Table 5). 81% of sites reported processing endoscopes in a separate room from where endoscopy is performed. 94% of nurses wear PPE including goggles while 63% of scope processing rooms have proper ventilation. All sites report their cleaning is done manually. 44% of sites have storage cabinets for their endoscopes. One site has an automated drying cabinet.

**TABLE 5:** How endoscopes are cleaned and processed. ✓ designates cleaning and disinfecting systems in the reporting sites.

Country	Endoscopes processed by nurses	Cleaning is done manually	Processing in a separate room	Nurses wear PPE during processing	Processing room has ventilation	Scope storage cabinets
American Samoa	✓	✓	✓	✓	✓	
Cook	✓	✓	✓	✓	✓	
FSM Chuuk	✓	✓	✓	✓		
FSM Pohnpei	✓	✓	✓	✓	✓	
Fiji Lautoka	✓	✓		✓		
Fiji Labasa	✓	✓	✓	✓		✓
Fiji CWM	✓	✓	✓	✓	✓	✓
Fiji Private	✓	✓	✓	✓	✓	
Kiribati	✓	✓		✓	✓	
Marshall Islands	✓	✓		✓	✓	✓
Palau	✓	✓	✓	✓	✓	
Samoa	✓	✓	✓	✓	✓	
Solomon Islands	✓	✓	✓	✓	✓	✓*
Tonga	✓	✓	✓	✓		
Tuvalu	✓	✓	✓			
Vanuatu	✓	✓	✓	✓		✓

* Denotes an automated drying cabinet.

Instruments were cleaned and disinfected with glutaraldehyde solution alone in 3 sites, with enzyme solution followed by glutaraldehyde in 7, with Matrix followed by Instrumax and glutaraldehyde by 1, dishwashing liquid and Savlon in 2 and Teepol (sodium alkyl benzene sulfonate, sodium ether sulfate, and an alcohol ethoxylate) followed by glutaraldehyde in 3. A number of sites obtain processing chemicals from 2 or 3 sources including through the MOH budgets, the National Medical Supply Store, and donations as the most common sources.

### 3.5 *Helicobacter pylori* Testing and Pathology Specimen Handling

Methodology in testing for *H. pylori* varied (Table 6). The majority (63%) of survey respondents send pathology specimens out of the country (overseas) by contract arrangement. Six (37%) of sites process their pathology specimens in country at a public lab. One site (Pohnpei in FSM) uses a hybrid system of in‐country specimen processing and telepathology review provided by a Japanese pathologist.

**TABLE 6:** Method used for testing for *Helicobacter pylori* . ✓ designates the type of *H. pylori* test used in country. Commercially available test kits denote a rapidly diagnose *H. pylori* kit that uses a gastric biopsy. Commercial kits used include Pronto Dry and Clotests.

Country	Commercially available test kits	Rapid urease test	Histopathology Geimsa stain	*H. pylori* serology test	Stool antigen	Urease breath test
American Samoa	✓				✓	
Cook						
FSM Chuuk	✓	✓		✓		
FSM Pohnpei		✓		✓		
Fiji Lautoka			✓			✓
Fiji Labasa	✓		✓	✓		
Fiji CWM	✓	✓	✓			
Fiji Private						
Kiribati	✓		✓			
Marshall Islands	✓					
Palau	✓					
Samoa	✓	✓	✓			
Solomon Islands	✓	✓	✓			
Tonga		✓				
Tuvalu				✓		
Vanuatu	✓					

### 3.6 Endoscopic Procedure Reporting and Telehealth Availability

Five of 16 sites use a computer‐based endoscopy reporting system. Four out of five use an internet‐based endoscopy program such as Provation, Scopeside, or Fijiscope. One site, American Samoa, has an endoscopy report template linked to its CareVue electronic health record. In Solomon Islands, a Microsoft Word template was created for reporting and it is stored on the endoscopy unit computer.

Eleven out of 16 sites report access to telehealth. The most common location of telehealth equipment is in hospitals as reported by 10 of 16 sites. One site has access to telehealth only in their clinic. Four sites have access to telehealth in both hospital and office or clinic locations. Multiple modalities are used for telehealth with all respondents employing multiple programs such as Zoom (69%), Facebook Messenger (69%), Viber (50%), WebEx Skylink, and WhatsApp.

### 3.7 Barriers and Challenges

Of the reported barriers toward establishing and sustaining endoscopy services, having functioning endoscopy equipment (98%), the cost of maintaining endoscopic equipment (scopes, processors, and monitors) (88%), and lack of training for both nurses as well as doctors, and lack of a designated budget allowing for the purchasing of equipment and consumable supplies were the most common. Other perceived barriers are shown in Table S2.

## 4 Discussion

The present study represents the first comprehensive assessment of endoscopy capacity across 11 PIC, covering 16 distinct sites. The findings indicate that while most PIC possess the ability to perform endoscopy, there is significant disparity in the availability of human and material resources. Common barriers to providing endoscopy services include limited access to functioning equipment and consumables, alongside the high cost of service and maintenance of endoscopes.

The total number of practitioners performing endoscopy (both surgeons and physicians) across the PIC is 58, equating to a rate of 2.1 per 100 000 population. This is considerably lower than neighboring countries like Australia and New Zealand [13, 14]. Despite being higher than other low‐resource regions, the overall data might present a misleadingly positive picture. In four countries—Fiji, Samoa, Tonga, and the Solomon Islands—there is a relatively large clinical workforce of endoscopists, nurses, and trainees. This workforce has benefitted from intensive local training and support from partnerships with ANZGITA and the Fiji WGO training center, leading to the development of local champions who continue to support training and service delivery. Fiji, in particular, has numerous endoscopists who have benefitted from training at the WGO‐designated training hub and postgraduate training at the Fiji National University.

Conversely, in four countries—Tuvalu, Pohnpei, the Republic of Marshall Islands, and Palau—only a single endoscopist is available, with no trainees. These countries have not received regular training opportunities or development assistance and have low numbers of surgeons and physicians per capita. This situation poses a risk of service loss if practitioners are reassigned or migrate. The region as a whole faces a shortage of healthcare practitioners, with multitasking cited as a barrier to endoscopy performance [4, 15, 16]. Dedicated nurses for endoscopy are available at all sites, but 94% are frequently involved in other clinical care areas. Fiji's CWMH has developed a successful model by empowering a dedicated endoscopy nurse to manage the service, a model that may not be feasible in regions with limited nursing staff.

The overall rates of colonoscopy are lower than gastroscopy throughout the region, with only two sites performing similar numbers of both procedures. The highest annual rate of gastroscopy was in Pohnpei (13.3 per 1000) and the lowest in Vanuatu (0.1 per 1000). The number of endoscopy procedures is influenced by individual site skills, population catchment, referral patterns, and the availability of consumables. For example, the Solomon Islands have a lower rate despite performing an average of 30 cases per month. This is an access issue, as 80% of the population lives in rural outer islands, requiring travel to the National Referral Hospital in Honiara on Guadalcanal [17]. Similar challenges are faced in Vanuatu, the Republic of Marshall Islands, and Kiribati. Funding and support sources also affect service rates. In American Samoa, a US territory, and the Federated States of Micronesia, a US Freely Associated State, funding for healthcare is through Administrative services and Compacts of Free Association through the US Department of Interior's Office of Insular Affairs. This has resulted in higher rates of health expenditure per capita in the US‐Affiliated Pacific Islands as opposed to non‐US‐Affiliated Pacific Islands [18]. The higher expenditure in the US‐Affiliated Pacific Islands does not necessarily reflect the addition of endoscopic services. While the authors do expect health expenditures to go up as countries take over running their own endoscopy units, these costs can be offset by savings from a reduction in overseas medical referrals. A cost‐benefit analysis from in‐country endoscopy services is currently being conducted in Solomon Islands.

The authors note that within the Pacific Islands procedure availability and indications for using it tend to be centralized which poses access limitations due to geography in addition to skills and training. A few countries have multiple sites where endoscopy is performed. This expansion of serves to outer islands may contribute to increased access.

This study also found that there is broad skill capacity for performing simple therapeutic techniques during endoscopy, but this is often limited by the availability of consumables. Only 50% of sites had access to a diathermy unit, limiting thermal therapy for treating bleeding from peptic ulcer disease, which is a common disease due to the high prevalence of *H. pylori* and easy access to Non‐Steroidal anti‐inflammatory medications [19, 20].

Advanced endoscopic procedures are primarily conducted in Fiji and the Solomon Islands, with the Colonial War Memorial Hospital in Fiji being the only site performing ERCP. Training efforts have focused heavily on these two countries, with clinicians benefiting from brief fellowship training in Australia, leading to the development of advanced therapeutic endoscopy and ERCP locally. The availability of supplies such as esophageal stents, balloons, and PEG kits remains a significant challenge, heavily reliant on donations and industry partnerships.

Sustainable capacity is hampered by equipment in addition to consumables. Twelve sites had fewer than six endoscopes, and many of these were not operational. Fiji and the Solomon Islands accounted for 38% of all endoscopes available in the region, but only 50% were functional due to high turnover use and maintenance challenges. Most sites rely on donations, with many endoscopes already extensively used, making them prone to damage. Maintenance often requires sending equipment overseas, a costly and time‐consuming process.

Cleaning and disinfection of endoscopes are performed manually at most sites, with only one facility having semi‐automated equipment. Nearly a third of sites reported inadequate ventilation for disinfection, and only one site had an automated drying cabinet. Single‐use supplies are often reused to save costs, posing challenges for proper cleaning and sterilization, and impacting the local environment.

Novel solutions are needed to address isolation and resource limitations, such as centralized bulk purchasing of consumables, and developing regional maintenance hubs. Emerging markets like China and India may provide low‐cost endoscopes, but partnerships with industry and hospital networks will be necessary for a sustainable supply of equipment. One example of how regional cultural norms must be included in addressing cost challenges is *H. pylori* testing. Stool antigen tests are very inexpensive and economically make sense in a resource limited health system, however, the Pacific Island cultural attitude toward collecting stool has hindered its use and would require significant public education.

As more PICs establish endoscopic services, national as well as regional standards for certification should be developed. With 58 endoscopists in the region, consideration should be given to establishing a gastrointestinal endoscopy professional organization. Our study found a wide variation in endoscopy reporting systems. The addition of a standardized endoscopy reporting system with photodocumentation would allow the sharing of data across the Pacific region and would facilitate collecting regional cancer as well as gastrointestinal disease data.

The availability of telehealth connectivity at each responding site can assist with real‐time teaching as well as consultative services. Telehealth has been employed on a limited basis in Samoa and Palau where ANZGITA/FNU partners consult before, during, and after endoscopy. The addition of a formal service can assist in furthering endoscopic services and patient care and encourage within country connectivity.

The introduction of endoscopy services has contributed positively to patient care, although this study's intent was to assess capacity rather than outcomes. Increased surgical, oncological, and medical treatments have resulted from endoscopic diagnoses. Advanced surgical training is needed to treat cancers diagnosed by endoscopy, combining local and overseas training. Oncological protocols are in place for treating gastrointestinal cancers in some countries. Future analysis of endoscopy services and how they impact patient care in the Pacific Islands are planned including more detailed data on the demographics of endoscopists along with the type of experience and interventions based upon endoscopy findings.

Partnerships between organizations like FNU, WGO, and ANZGITA and PICs demonstrate how dedicated efforts can develop, improve, and sustain high‐quality services in low‐resource countries. Local partners need to work with the Ministry of Health on sustainable funding once services are established. The addition of endoscopy services aligns with the goal of Universal Health Coverage (UHC) supported by the WHO and the World Bank, aiming for all people to access quality health services without financial hardship [21].

In conclusion, our study confirms a broad and widespread capacity for endoscopy across the Pacific region. Despite challenges in skilled healthcare worker numbers, functioning equipment, and consumables, there is strong support for endoscopy services. Careful investment in training, healthcare funding, and partnership development is needed to ensure equity in endoscopy services across the PIC.

## Conflicts of Interest

The authors declare no conflicts of interest.

## Supporting information


**Figure S1.** Annual rates of upper and lower endoscopy per 1000 population performed at responding PIC sites.
**Table S1.** Disposable or single‐use endoscopy supplies. Note dilators include both Savary Gillard and single‐use balloon dilators. ✓ designates single‐use supplies used in country.
**Table S2.** Issues and barriers to establishing endoscopy programs.
