# Supplementary material for: A Quantitative Analysis of Human and Material Resources for Endoscopy Services in Pacific Island Countries
Source: JGH Open. 2024 Dec 12;8(12):e70068. doi: 10.1002/jgh3.70068 (PMC11636305; doi:10.1002/jgh3.70068)
Supplement: Supplementary file 1 — Figure S1. Annual rates of upper and lower endoscopy per 1000 population performed at responding PIC sites. Table S1. Disposable or single‐use endoscopy supplies. Note dilators include both Savary Gillard and single‐use balloon dilators. ✓ designates single‐use supplies used in country. Table S2. Issues and barriers to establishing endoscopy programs. [file JGH3-8-e70068-s001.docx]

**SUPPLEMENTARY FIGURES AND TABLES**

Figure s1: Annual rates of upper and lower endoscopy per 1000 population performed at responding PIC sites.


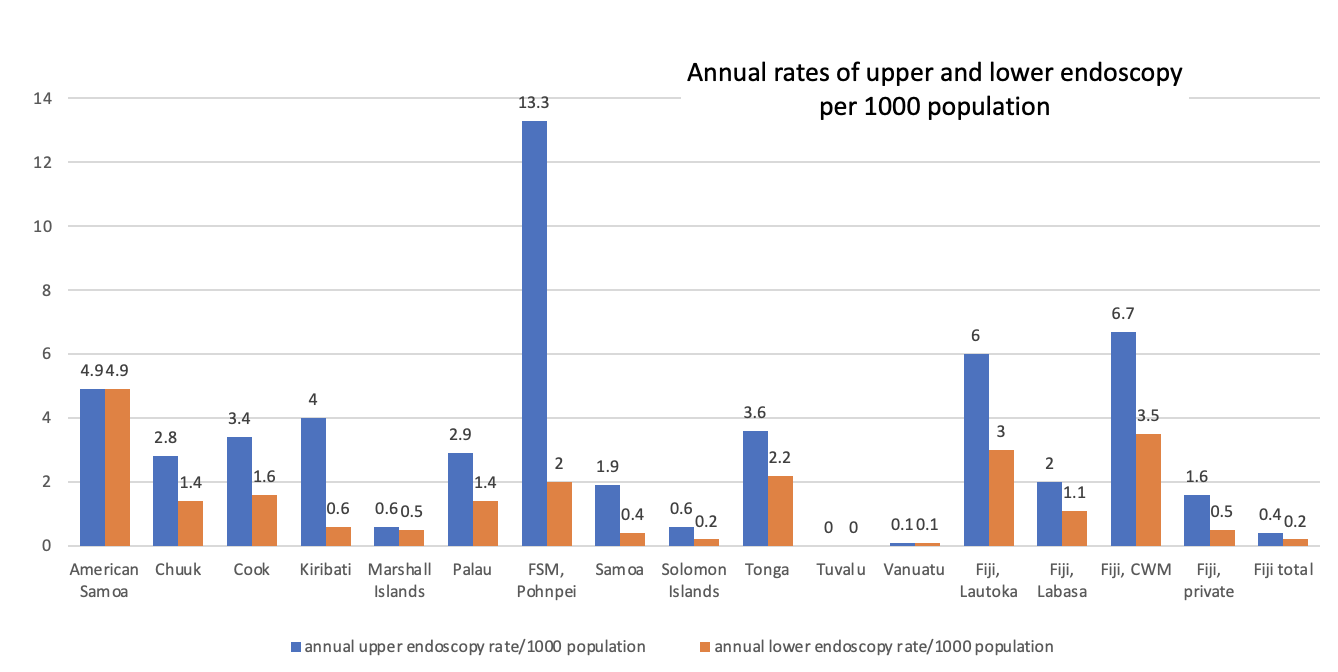


Table s1: Disposable or single use endoscopy supplies. Note dilators include both Savary Gillard and single use balloon dilators. ✓ designates single use supplies used in country

| **Country** | **Biopsy forcep** | **Brushes for cleaning** | **Brush for cytology** | **Injecting needle** | **Thermal probe** | **Dilators** | **Esophageal**  **stents** | **Colonic stents** | **Snares** | **Foreign body retrieval forceps** |
| --- | --- | --- | --- | --- | --- | --- | --- | --- | --- | --- |
| American Samoa | ✓ | ✓ |  | ✓ | ✓ |  |  |  | ✓ | ✓ |
| Cook | ✓ | ✓ | ✓ |  |  |  |  |  | ✓ | ✓ |
| FSM Chuuk | ✓ | ✓ |  | ✓ | ✓ | ✓ |  |  |  | ✓ |
| FSM Pohnpei | ✓ | ✓ | ✓ | ✓ |  | ✓ |  |  | ✓ | ✓ |
| Kiribati | ✓ | ✓ |  | ✓ |  | ✓ |  |  | ✓ | ✓ |
| Marshall Islands | ✓ | ✓ |  | ✓ |  |  |  |  | ✓ | ✓ |
| Palau | ✓ | ✓ |  |  |  |  |  |  | ✓ | ✓ |
| Samoa | ✓ | ✓ | ✓ | ✓ | ✓ |  |  |  | ✓ | ✓ |
| Solomon Islands | ✓ | ✓ |  | ✓ |  | ✓ | ✓ |  | ✓ | ✓ |
| Tonga | ✓ | ✓ |  | ✓ |  | ✓ |  |  | ✓ | ✓ |
| Tuvalu |  |  |  |  |  |  |  |  |  |  |
| Vanuatu | ✓ | ✓ |  | ✓ |  | ✓ |  |  | ✓ | ✓ |
| Fiji Lautoka | ✓ | ✓ |  | ✓ | ✓ | ✓ |  |  | ✓ | ✓ |
| Fiji Labasa | ✓ | ✓ |  | ✓ |  | ✓ |  |  | ✓ | ✓ |
| Fiji CWM | ✓ | ✓ | ✓ | ✓ | ✓ | ✓ | ✓ | ✓ | ✓ | ✓ |
| Fiji Private | ✓ | ✓ |  |  |  |  |  |  |  |  |

Table S2: Issues and barriers to establishing endoscopy programs

| Issue | Sites responding |
| --- | --- |
| Functioning Equipment | 15 (98%) |
| Cost of maintenance | 14 (88%) |
| Lack of training for Nurses | 12 |
| Lack of training for doctors | 11 |
| Lack of nursing staff | 10 |
| Cost of purchasing equipment: | 10 |
| Lack of designated funding | 10 |
| Lack of consumables | 10 |
| Lack of local pathology processing | 9 |
| Location availability | 9 |
| Time commitment | 8 |
| Lack of disinfectant | 8 |
| Lack of MOH support | 6 |
| Issues with electricity | 1 |
| Issues with water | 1 |
| Cost to patients | 1 |
